# Prevalence and determinants of sleep problems in cancer survivors compared to a normative population: a PROFILES registry study

**DOI:** 10.1007/s11764-024-01641-z

**Published:** 2024-07-24

**Authors:** Charles David, Sandra Beijer, Floortje Mols, Simone Oerlemans, Olga Husson, Matty P Weijenberg, Nicole PM Ezendam

**Affiliations:** 1 Department of Research and Development, Netherlands, Comprehensive Cancer Organisation , Godebaldkwartier 419, 3511 DT Utrecht, the Netherlands; 2 CoRPS-Center of Research on Psychological Disorders and Somatic diseases, Department of Medical and Clinical Psychology, Tilburg University, Tilburg, the Netherlands; 3 Division of Psychosocial Research and Epidemiology, Netherlands Cancer Institute, Amsterdam, the Netherlands; 4 Department of Medical Oncology, Netherlands Cancer Institute, Amsterdam, the Netherlands; 5 Department of Surgical Oncology, Erasmus Medical Center, Rotterdam, the Netherlands; 6 Department of Epidemiology, GROW-School for Oncology and Developmental Biology, Maastricht University, Maastricht, the Netherlands

**Keywords:** Cancer survivors, Sleep quality, Prevalence, Determinants of sleep quality

## Abstract

**Purpose:**

To (1) identify the prevalence of sleep problems in cancer survivors across cancer types and survivorship durations compared to a normative population and (2) determine demographic, clinical, lifestyle, and psychosocial determinants.

**Method:**

Cancer survivors diagnosed between 2008 and 2015 (*N* = 6736) and an age- and sex-matched normative cohort (*n* = 415) completed the single sleep item of the EORTC QLQ-C30: Have you had trouble sleeping? Participants who responded with “quite a bit”/ “very much” were categorized as poor sleepers. A hierarchical multinomial logistic regression was used to identify determinants of sleep problems.

**Result:**

The prevalence of sleep problems was higher in cancer survivors (17%) compared to the normative population (11%) (*p* < 0.001), varied across cancer types (10–26%) and did not vary based on survivorship duration. The full model showed that survivors who were female (adjusted odds ratio (AOR) 2.26), overweight (AOR 1.50), had one (AOR 1.25) and ≥ 2 comorbidities (AOR 2.15), were former (AOR 1.30) and current (AOR 1.53) smokers and former alcohol drinkers (AOR 1.73), had a higher level of fatigue (AOR 1.05), anxiety (AOR 1.14), depression (AOR 1.11), and cognitive illness perceptions (AOR 1.02), had a higher odds for sleep problems. Higher education compared to lower education (AOR 0.67), having a partner (AOR 0.69), and obesity compared to normal BMI (AOR 0.86) were protective to sleep problems as well as high physical activity before adjusting for psychological factors (AOR 0.91).

**Conclusion:**

Modifiable determinants of sleep problems include physical activity, fatigue, anxiety, depression, and illness perception.

**Implications for Cancer Survivors:**

Sleep problems after cancer deserve clinical attention. They may be improved by addressing modifiable lifestyle factors: increasing physical activity, stop smoking, and reducing alcohol consumption. As fatigue, depression, and illness perception seem related to sleep problems, lifestyle improvements may also improve these outcomes.

## Introduction

Sleep problems are a common problem in cancer survivors—individuals with a cancer diagnosis, extended treatment, and advanced cancer [1–4]. Sleep problems have been shown to be associated with increased distress, morbidity, reduced productivity (daytime functioning), and poor health-related quality of life (HRQoL) in cancer survivors [5–7].

The burden of sleep problems in cancer survivors is higher when compared to the general population affecting 20–70% of treated individuals [8–12] and varies across cancer types. Studies have demonstrated sleep problems in up to 17% of prostate cancer survivors, 47% of colorectal cancer survivors, 59% of ovarian cancer survivors, and 72% of endometrium cancer survivors [5, 13–15]. However, fewer studies have compared sleep problems among survivors of different cancer types, those being longer post diagnosis (> 5 years) and those receiving different types of cancer treatment [6, 8, 9]. Additionally, a limitation in many studies has been a small sample size when investigating the prevalence across cancer types [2, 14, 16], and only few studies included a matched normative population for comparison with the general population’s prevalence [4, 8, 9].

Various determinants have been associated with sleep problems in cancer survivors. Demographic factors included lower age, female sex, higher education, and not being able to work to be associated with sleep problems [1, 3, 6, 8, 9, 11, 14, 17–20]. Clinical factors included higher body mass index (BMI), cancer type and higher stage during diagnosis, type of treatment (chemotherapy and radiotherapy), shorter time since diagnosis, and the presence of comorbidities [5, 6, 8, 9, 14, 19, 20]. Lifestyle factors such as smoking, drinking alcohol, and not being physically active have shown to be significantly associated with sleep problems [8, 9, 18]. Lastly, various psychosocial factors including anxiety, depression, and fatigue have been negatively associated with sleep problems among cancer survivors [8, 9, 13, 14, 21–23]. Because literature shows that illness perception is associated with fatigue, anxiety, and depression among cancer survivors [24–26], it is also likely to be related to sleep problems.

Although these associations are well described, there is a lack of evidence that addresses all these determinants together with several types of cancer in one single study. Investigating both demographic and clinical data together with modifiable lifestyle and psychosocial factors will not only give an indication which cancer survivors are at risk of sleep problems, it will also give an indication for the development of interventions. Furthermore, it is important to assess all possible factors in a single study to address the effect of potential confounding.

This study aimed to assess the prevalence of sleep problems in cancer survivors compared to an age- and sex-matched normative population, among survivors with different cancer types (colon, rectum, prostate, ovarian, thyroid, endometrium, Hodgkin and non-Hodgkin lymphoma, chronic lymphocytic leukemia, melanoma, multiple myeloma, and basal/squamous cell carcinoma) and according survivorship duration post diagnosis. The second aim was to identify various demographic, clinical, lifestyle, and psychosocial determinants that were associated with sleep problems. The results from this study aim to increase insights into cancer survivors who have a higher risk of sleep problems to create awareness among medical professionals.

## Methods

### Study design

This study is a secondary quantitative data analysis of a cross-sectional population-based sample from the Patient Reported Outcome Following Initial treatment and Long-term Evaluation of Survivorship (PROFILES) registry in The Netherlands. PROFILES is a registry facilitating data collection on patient-reported outcomes from cancer survivors. Its main objectives are psychosocial risk and outcome assessment, understanding biological and behavioral factors associated with cancer treatment outcomes, and evaluating cancer survivors physical and psychological care needs [27]. Patients for this study were included between May 2008 and April 2015.

### Study population and selection procedure

Cancer survivors were selected from the Netherlands Cancer Registry, which contains data of all newly diagnosed cancer patients in the Netherlands since 1989. Cancer survivors (*N* = 10,304) who were diagnosed between 2008 and 2015with endometrium cancer, colorectal cancer, Hodgkin and non-Hodgkin lymphomas, multiple myeloma, chronic lymphocytic leukemia, thyroid cancer, prostate cancer, ovarian and borderline ovarian cancer, basal/squamous cell carcinoma, and melanoma in selected hospitals were eligible [27, 28]. To adopt a generalized few across cancers, we selected as wide a variety of tumor types as possible such as common tumor types and rare tumor types, solid tumors and hematologic tumors, and tumors that occur only in men or women vs. tumors that occur in both. These cancer survivors were asked to participate in the study by an invitation letter from their former/treating physician. Participants signed the informed consent forms and completed the questionnaires (digital or by paper and pencil) [27]. Inclusion criteria were the capability of reading and writing the Dutch language and completing self-reported questionnaires without extensive assistance [27]. People who died or emigrated before the start of the studies were excluded (according to data from the hospital of diagnosis and the Dutch municipal record database) [28]. Non-participation in the study had no consequence on the treatment or any follow-up care, and there was no risk/harm to the participants [27]. Ethical approval was obtained from a local certified medical ethics committee.

The PROFILES registry also contained a reference cohort of adult individuals (*N* = 1557) from the general Dutch population [27]. This data was generated by CentERdata (www.centerdata.nl), a Dutch research institute, and comes from the LISS panel which is part of CentERdata NL (https://www.centerdata.nl/liss-panel). The LISS panel aims to provide a good representation of the Dutch population for research purposes. The panel is probability based and uses the sample of households from the population register of Statistics Netherlands (CBS). Respondents were asked whether they have had cancer in the past, and if so, they were removed from the dataset. On an annual basis, the participants of this CentERpanel were asked to complete various questionnaires similar to the ones used in PROFILES [27]. Participants from this reference cohort were matched overall and with each cancer type on age and sex to create normative populations.

### Measurement instruments

#### Sleep problems (outcome)

Sleep problems were measured using a single item of the European Organization for Research and Treatment of Cancer Quality of Life Questionnaire (EORTC QLQ)-C30: “During the past week—Have you had trouble sleeping?” Participants could answer by selecting one of the four options: not at all, a little, quite a bit, and very much. For the analyses, answers were recoded into three categories: survivors with no sleep problems (people who did not have any trouble sleeping), moderate sleep problems (people who had little trouble sleeping), and many sleep problems (people who had quite a bit and very much trouble sleeping) [29]. This aligned with the threshold for clinical cut off that was identified in the literature by Giesinger et al. [30, 31], differentiating between poor vs. good/moderate sleep quality. Furthermore, the EORTC QLQ-C30 is a well-validated and reliable questionnaire to assess HRQoL [32]. The single sleep item of the EORTC QLQ-C30 appeared in previous research as a sufficient measure to study sleep at group level, and we found in a previous study that the sleep item was highly correlated (*r* = 0.71) with the total score of the Pittsburgh Sleep Quality Index (PSQI) [33, 34].

#### Determinants of sleep quality

The determinants of interest in this study were the variables from the literature that have previously shown to be associated with sleep problems [1–3, 5, 6, 8, 9, 14, 15, 18].

#### Demographic

Demographic factors included age, sex, marital status, level of education, and working status. The variable sex was categorized as male and female. Marital status was categorized as having a partner (married or cohabiting) and not having a partner (divorced/separated/widowed/never married/never cohabited). Level of education was categorized as low (less than high school), secondary (high school or vocational), and high (bachelor’s or master’s education). Current working status was categorized as yes or no.

#### Clinical

Data for cancer type, stage (tumor, node, metastasis (TNM) except for lymphomas, multiple myeloma (MM), and chronic lymphocytic leukemia (CLL)), primary treatment (surgery, radiotherapy, chemotherapy, and systemic and hormonal therapy), time since diagnosis (< 2, ≥ 2 to** < **3, ≥ 3 to** < **5, and ≥ 5 years), and comorbidities (0, 1, ≥ 2 comorbidities)) were obtained from the Netherlands Cancer Registry.

#### Lifestyle

Data on smoking (no, former, yes), alcohol use (no, former, yes), physical activity (hours of moderate to vigorous physical activity (MVPA) per week), and body height and weight (body mass index (BMI), kg/m^2^) were self-reported. Physical activity was recorded using the European Perspective Investigation of Cancer, EPIC physical activity questionnaire wherein MVPA was derived using metabolic equivalents (MET), the ratio of a person’s resting metabolic rate compared to the metabolic rate during a specific activity. A MET score of 1 represents the amount of energy used at rest while a score of 3–6 is categorized as moderate and any activity above 6 MET as vigorous physical activity [35]. For the analyses, we categorized MVPA/week into tertiles (MVPA/week 0–6 = lowest, 6.01–12.05 = middle, and 12.6––49 = highest).

#### Psychosocial

Fatigue was assessed using the Fatigue Assessment Scale (FAS). The FAS is a 10-item scale that evaluates chronic fatigue as a unidimensional construct. The items are directed towards the usual feeling of the participant about how fatigue affects their functioning. All ten items are scored on a 5-point scale never/sometimes/regularly/often/always. The total score is the sum score of all items ranging from 10 to 50; a total score of ≥ 22 indicates fatigue [36]. The FAS has shown high reliability, content validity, and internal consistency (Cronbach’s *α* = 0.87), in the Dutch population [37].

Anxiety and depression were assessed using the Hospital Anxiety and Depression scale (HADS). The HADS is a 14-item self-reported questionnaire frequently used in oncology and other physical health settings to screen participants’ emotional distress. Participants answer questions about anxiety and depressive symptoms (seven items each) on a scale of 0–3. A higher score is associated with a higher level of anxiety and depression (0–7 = normal, 8–10 = borderline anxiety or depressive symptoms) [38]. The cutoff sum score of ≥ 15 on the full scale has shown a sensitivity of 80%, a specificity of 76%, and a positive predictive value of 41% when compared to the general health questionnaire (GHQ 28) and Rotterdam symptom checklist (RSCL) [39].

Illness perception was measured using the Brief illness perception questionnaire (BIPQ). The BIPQ is an eight-item self-reported questionnaire used to measure cognitive (5 items; consequence, timeline, personal control, treatment control, and identity) and emotional representations (2 items; concern and emotion) of the illness and illness comprehensibility (1 item). All items are scored on a 1–10 linear point scale with higher scores indicating a negative perception [40]. The scale has shown good concurrent and predictive validity with a good ability to access changes over time [40].

### Statistical analysis

The demographic, clinical, lifestyle, and psychosocial characteristics of the total population, norm population, and non-respondents were described using means and standard deviations (SD) for normally distributed and medians and interquartile ranges (IQR) for non-normally distributed continuous determinants. Data for categorical variables were presented as frequencies and percentages (presented in Table 4). The sample size that answered the EORTC QLQ-C30 sleep question (Q11) was used to match with the normative population. We matched the normative population with overall cancer survivors based on age and sex, after removing participants who have had cancer. Cancer survivors and normative population samples were grouped into a strata of gender (male, female) and age categories (≤ 45, > 45 to ≤ 50, > 50 to ≤ 55, > 55 to ≤ 60, > 60 to ≤ 65, > 65 to ≤ 70 and > 70), and matched per stratum according to the most limiting ratio of 1:13. This resulted in a sub sample of 415 age- and sex-matched participants from the normative population.

To answer the first research question on the prevalence of sleep problems, the proportion of participants (frequencies and percentage) having no, moderate, and many sleep problems was described for the cancer survivors and for the normative population, separately for the total cancer population and per cancer type (presented in Table 1).

**Table 1:** Comparison of prevalence of sleep problems between cancer survivors of specific cancer types with a (for each cancer type) age- and sex-matched normative population. Multinomial logistic regression analyses with the normative and no sleep problems as reference group

	*N*	Sleep problems			Univariate OR (95% CI) cancer survivors *No sleep problems (ref.)*	
		No	Moderate	Many	Moderate	Many
Cancer type (all) *n *(age- and sex-matched normative)*	6736 *415*	3950 (59%) *243 (59%)*	1646 (24%) *126 (30%)*	1140 (17%) *46 (11%)*		
Colorectal *n*	2557	1485 (58%)	653 (26%)	419 (16%)	0.80 (0.62–1.04)	1.79 (1.20–2.60)
	*325*	*191 (59%)*	*104 (32%)*	*30 (9%)*	*Ref*	*Ref*
Prostate *n*	686	429 (63%)	156 (23%)	101 (15%)	0.89 (0.56–1.42)	1.72 (0.86–3.49)
	*114*	*74 (65%)*	*30 (26%)*	*10 (9%)*	*Ref*	*Ref*
Borderline ovarian *n*	83	52 (63%)	20 (24%)	11 (13%)	0.62 (0.35–1.11)	1.27 (0.58–2.77)
	*269*	*151 (56%)*	*93 (35%)*	*25 (9%)*	*Ref*	*Ref*
Ovarian *n*	354	151 (43%)	111 (31%)	92 (26%)	1.24 (0.86–1.78)	3.02 (1.89–4.84)
	*267*	*149 (56%)*	*88 (33%)*	*30 (11%)*	*Ref*	*Ref*
Thyroid *n*	300	168 (56%)	76 (25%)	56 (19%)	0.87 (0.62–1.20)	1.85 (1.23–2.78)
	*576*	*339 (59%)*	*176 (31%)*	*61 (11%)*	*Ref*	*Ref*
Endometrial *n*	217	116 (53%)	60 (28%)	41 (19%)	0.94 (0.58–1.52)	1.85 (0.97–3.52)
	*146*	*84 (58%)*	*46 (32%)*	*16 (11%)*	*Ref*	*Ref*
Hodgkin lymphoma *n*	209	130 (62%)	46 (22%)	33 (16%)	0.88 (0.61–1.28)	1.96 (1.24–3.10)
	*840*	*550 (65%)*	*219 (26%)*	*71 (8%)*	*Ref*	*Ref*
NHL *n*	1121	644 (58%)	273 (24%)	203 (18%)	0.99 (0.73–1.35)	2.56 (1.60–4.09)
	*277*	*179 (65%)*	*76 (27%)*	*22 (8%)*	*Ref*	*Ref*
CLL *n*	281	162 (58%)	56 (20%)	63 (22%)	0.78 (0.54–1.13)	3.91 (2.39–6.40)
	*419*	*272 (65%)*	*120 (29%)*	*27 (6%)*	*Ref*	*Ref*
Multiple myeloma *n*	256	143 (56%)	58 (23%)	54 (21%)	0.75 (0.50–1.13)	3.00 (1.69–5.30)
	*252*	*152 (60%)*	*81 (32%)*	*19 (8%)*	*Ref*	*Ref*
BCC/PCC *N*	674	470 (70%)	137 (20%)	67 (10%)	0.62 (0.40–0.94)	1.31 (0.63–2.73)
	*131*	*83 (63%)*	*39 (30%)*	*9 (7%)*	*Ref*	*Ref*

*Univariate* odds ratio (OR) based on multinomial logistic regression analyses

*BCC/PCC* basal/squamous cell carcinoma,* NHL* non-Hodgkin Lymphoma,* CLL* chronic lymphocytic leukemia

^*^Chi square test to assess the difference between sleep quality of cancer survivors and that of the norm population: effect size = 0.127, *p* < 0.001

To answer the second research question on the determinants of sleep problems, we first descriptively assessed differences in demographic, clinical, lifestyle, and psychosocial characteristics of survivors with no, moderate, and many sleep problems. Differences in these characteristics between sleep problem groups were assessed using analysis of variance (ANOVA) and Pearson chi square for continuous and categorical variables respectively (presented in Table 2).

**Table 2:** Demographic, lifestyle, clinical, and psychosocial characteristics of study population for survivors with no, moderate, and many sleep problems (n%). Univariate ORs based on multinomial univariable logistic regression, with sleep problems of cancer survivors as dependent variable (no sleep problems is the reference), *N* = 3979

Determinants, N%	Sleep problems			Univariate Odds ratio (95% CI) *No sleep problems ref.*		*p* value
	No *n* = 2318 (58%)	Moderate *n* = 982 (25%)	Many *n* = 679 (17%)	Moderate	Many	
Age (mean, ± SD in years)	65.6 (± 12.4)	65.3 (± 12.0)	66.6 (± 11.7)	0.99 (0.99–1.00)	1.00 (1.00–1.01)	0.085
Sex						** < 0.001**
Male	1444 (62%)	447 (46%)	278 (41%)	Ref	Ref	
Female	874 (38%)	535 (54%)	401 (59%)	**1.97** (1.70–2.29)**	**2.38** (2.00–2.83)**	
Education						** < 0.001**
Low (less then high school)	385 (17%)	172 (18%)	129 (19%)	Ref	Ref	
Secondary (high school)	1370 (60%)	594 (61%)	435 (65%)	0.97 (0.79–1.19)	0.94 (0.75–1.18)	
High (bachelors or masters)	543 (23%)	205 (21%)	99 (15%)	0.84 (0.66–1.07)	**0.54** (0.40–0.72)**	
Work status						** < 0.001**
No	1710 (76%)	736 (78%)	568 (84%)	Ref	Ref	
Yes	546 (24%)	212 (22%)	97 (15%)	0.90 (0.75–1.08)	**0.53** (0.42–0.67)**	
Marital status						** < 0.001**
Divorced/separated/widowed	443 (19%)	234 (24%)	216 (32%)	Ref	Ref	
Having a partner	1857 (81%)	739 (76%)	455 (68%)	**0.75** (0.62–0.90)*	**0.50** (0.41–0.60)**	
Smoking						** < 0.001**
No	526 (31%)	275 (37%)	205 (41%)	Ref	Ref	
Former	981 (57%)	395 (52%)	231 (46%)	**0.77** (0.63–0.92)*	**0.60** (0.48–0.74)**	
Yes	214 (12%)	83 (11%)	64 (13%)	**0.74** (0.55–0.99)*	0.76 (0.55–1.05)	
Alcohol						** < 0.001**
No	358 (22%)	175 (25%)	175 (37%)	Ref	Ref	
Former	128 (8%)	47 (7%)	51 (11%)	0.75 (0.51–1.09)	0.81 (0.56–1.18)	
Yes	1116 (70%)	470 (68%)	249 (52%)	0.86 (0.69–1.06)	**0.45** (0.36–0.57)**	
Physical activity (MVPA/week)						**0.002**
Lowest (0–6)	594 (34%)	266 (35%)	217 (43%)	Ref	Ref	
Middle (6.01–12.05)	542 (31%)	249 (33%)	149 (30%)	1.02 (0.83–1.26)	**0.75** (0.59–0.95)** **0.62**	
Highest (12.06–49)	594 (34%)	240 (32%)	135 (27%)	0.90 (0.73–1.11)	(0.48–0.79)**	
BMI						**0.004**
< 18.5	25 (1%)	15 (2%)	17 (3%)	1.43 (0.74–2.74)	**2.21** (1.17–4.16)*	
18.5–24.9	837 (37%)	351 (36%)	257 (39%)	Ref	Ref	
25–29.9	1054 (46%)	426 (44%)	257 (39%)	0.96 (0.81–1.14)	**0.79** (0.65–0.96)*	
≥ 30	369 (16%)	175 (18%)	131 (19%)	1.13 (0.90–1.40)	1.15 (0.90–1.47)	
Tumor type						0.297
Colon/rectum	1485 (64%)	653 (67%)	419 (62%)	Ref	Ref	
Thyroid	168 (7%)	76 (8%)	56 (8%)	1.02 (0.77–1.36)	1.18 (0.85–1.62)	
Hodgkin lymphoma	102 (4%)	40 (4%)	22 (3%)	0.89 (0.61–1.30)	0.76 (0.47–1.22)	
NHL	381 (16%)	149 (15%)	113 (17%)	0.88 (0.72–1.09)	1.05 (0.82–1.33)	
CLL	94 (4%)	35 (4%)	34 (5%)	0.84 (0.56–1.26)	1.28 (0.85–1.92)	
Multiple myeloma	88 (4%)	29 (3%)	35 (5%)	0.74 (0.48–1.15)	1.40 (0.93–2.11)	
Tumor stage						0.765
I	649 (31%)	262 (29%)	179 (29%)	Ref	Ref	
II	710 (34%)	300 (33%)	206 (33%)	1.04 (0.85–1.27)	1.05 (0.83–1.31)	
III	539 (26%)	254 (28%)	163 (27%)	1.16 (0.94–1.43)	1.09 (0.86–1.39)	
IV	195 (9%)	88 (10%)	65 (11%)	1.11 (0.83–1.49)	1.20 (0.87–1.67)	
Primary treatment						
No/wait and see/watchful waiting	126 (5%)	47 (5%)	50(7%)	0.87 (0.62–1.23)	1.38 (0.98–1.94)	0.079
Yes	Ref			Ref	Ref	
Surgery						0.093
No	683 (29%)	260 (26%)	211 (31%)	Ref	Ref	
Yes	1635 (71%)	722 (74%)	468 (69%)	1.16 (0.98–1.37)	0.92 (0.76–1.11)	
Systemic						0.386
No	1383 (60%)	594 (60%)	425 (63%)	Ref	Ref	
Yes	935 (40%)	388 (40%)	254 (37%)	0.96 (0.82–1.12)	0.88 (0.74–1.05)	
Radiation						0.928
No	1549 (67%)	663 (68%)	455 (67%)	Ref	Ref	
Yes	769 (33%)	319 (32%)	224 (33%)	0.96 (0.82–1.13)	0.99 (0.82–1.18)	
Hormonal						0.430
No	2314 (99%)	978 (99%)	678 (99%)	Ref	Ref	
Yes	4 (0.1%)	4 (0.1%)	1 (0.1%)	2.36 (0.59–9.47)	0.85 (0.09–7.64)	
Time since diagnosis						0.478
< 2 years	285 (12%)	121 (12%)	97 (14%)	Ref	Ref	
≥ 2 to < 3 years	492 (21%)	198 (20%)	158 (23%)	0.94 (0.71–1.23)	0.93 (0.69–1.25)	
≥ 3 to < 5 years	496 (21%)	208 (21%)	135 (20%)	0.98 (0.75–1.28)	0.79 (0.58–1.07)	
≥ 5 years	1047 (45%)	455 (46%)	289 (43%)	1.01 (0.79–1.29)	0.80 (0.61–1.04)	
Comorbidities						** < 0.001**
No	698 (32%)	208 (22%)	87 (13%)	Ref	Ref	
1	672 (31%)	280 (30%)	165 (25%)	**1.39** (1.13–1.72)*	**1.96** (1.48–2.60)**	
≥ 2	808 (37%)	442 (48%)	397 (61%)	**1.83** (1.51–2.22)**	**3.94** (3.06–5.07)**	
Fatigue (mean, ± SD)	19.0 (± 6.2)	22.3 (± 6.6)	26.2 (± 8.2)	**1.07** (1.06–1.09)**	**1.14 (**1.13–1.16)**	** < 0.001**
Distress (mean, ± SD)						
Anxiety	3.4 (± 3.2)	5.2 (± 3.5)	7.4 (± 4.2)	**1.16 (**1.13–1.18)**	**1.31** (1.28–1.35)**	** < 0.001**
Depressive symptoms	3.4 (± 3.3)	4.5 (± 3.5)	6.6 (± 4.2)	**1.10** (1.07–1.12)**	**1.24** (1.21–1.27)**	** < 0.001**
Illness perception						** < 0.001**
Cognitive	23.7 (± 8.2)	25.3 (± 8.4)	27.3 (± 8.7)	**1.02** (1.01–1.03)**	**1.05** (1.04–1.06)**	
Emotional	6.5 (± 4.4)	8.1 (± 4.7)	10.1 (± 5.1)	**1.05** (1.03–1.06)**	**1.11** (1.09–1.13)**	**< 0.001**

*NHL* non-Hodgkin lymphoma,* CLL* chronic lymphocytic leukemia

*N* = 3979 after excluding patients that did not report data on demographic, clinical, lifestyle, and psychosocial factors

^*^p-value < 0.05, **p-value < 0.001

Univariable analyses (*N* = 3979) were conducted to identify which determinants were associated with moderate and many sleep problems. Subsequently, in a hierarchical multinomial logistic regression, four nested models were analyzed including blocks of determinants that were significant in the univariable analyses (*N* = 3979). Multiple imputation was used to impute data on participants who had missing data on any of the determinants [41].This assured that the same patients were analyzed in each step of the hierarchical regression analysis. The underlying assumptions for a multinomial regression were checked and not violated. The following blocks were entered consecutively: demographic (age, sex, education, working, and marital status), clinical (BMI, cancer type, cancer stage, primary treatment, time since diagnosis, and number of comorbidities), lifestyle (smoking, alcohol, and physical activity), and psychosocial determinants (fatigue, anxiety and depression, and illness perception). The order in which the different blocks were entered in the hierarchical regression analysis was based on the fact that we first entered sociodemographic background variables, then the clinical background variables, then lifestyle (determinants that are modifiable), and ultimately, the psychosocial variables that are most closely related to the outcome and may thus be interpreted as an outcome cluster. These were entered as the final step because one would otherwise over-adjust. The results were reported as adjusted odds ratios (AOR) with 95% CI accompanied by McFadden’s pseudo *R*^2^ values for all models [42], to understand the explained variability and improvement from the null model to the fitted model (presented in Table 3). The descriptive and univariate multinomial logistic regression analyses were done using Statistical Analysis Software (SAS) version 9.4 and while the hierarchical logistic regression analyses were done using STATA version 17 since SAS did not report pseudo *R*^2^ for each model separately. Statistical significance was denoted by a *p*-value of ≤ 0.05 for two-sided tests.

**Table 3:** Hierarchical multinomial logistic regression of demographic, clinical, lifestyle, and psychosocial determinants, with sleep problems as dependent variable (no sleep problems is the reference) (*N* = 3979)

Determinants	Model 1 (*N* = 3,979) demographic		Model 2 (*N* = 3979) demographic + clinical		Model 3 (*N* = 3979) demographic + clinical + lifestyle		Model 4 (*N* = 3979) demographic + clinical + lifestyle + psychosocial	
	Moderate	Many	Moderate	Many	Moderate	Many	Moderate	Many
Demographic								
Sex								
Male	Ref	Ref	Ref	Ref	Ref	Ref	Ref	Ref
Female	2.66 (2.50–2.82)**	1.38 (1.29–1.48)**	2.87 (2.70–3.05)**	1.49 (1.39–1.60)**	3.05 (2.85–3.27)**	1.62 (1.50–1.76)**	3.01 (2.79–3.25)**	2.26 (2.06–2.48)**
Education Low (less then high school)	Ref	Ref	Ref	Ref	Ref	Ref	Ref	Ref
Secondary (high school)	1.18 (1.08–1.28)*	0.68 (0.62–0.74)**	1.17 (1.07–1.28)**	0.76 (0.65–0.78)**	0.93 (0.85–1.02)	0.81 (0.74–0.89)*	0.97 (0.88–1.07)	0.95 (0.85–1.05)
High (bachelors or masters)	1.45 (1.31–1.61)**	0.63 (0.56–0.70)**	1.50 (1.35–1.67)**	0.68 (0.61–0.77)**	1.39 (1.24–1.56)**	0.81 (0.72–0.91)*	1.59 (1.41–1.79)**	0.67 (0.58–0.76)**
Work status								
No	Ref	Ref	Ref	Ref	Ref	Ref	Ref	Ref
Yes	0.58 (0.54–0.63)**	1.28 (1.19–1.38)**	0.73 (0.67–0.79)**	1.48 (1.37–1.60)**	0.57 (0.53–0.62)**	1.34 (1.24–1.46)**	0.51 (0.47–0.56)**	1.04 (0.95–1.14)
Marital status								
Divorced/separated/widowed	Ref	Ref	Ref	Ref	Ref	Ref	Ref	Ref
Having a partner	0.36 (0.34–0.38)**	0.58 (0.54–0.63)**	0.37 (0.35–0.39)**	0.56 (0.52–0.60)**	0.33 (0.31–0.36)**	0.52 (0.48–0.56)**	0.37 (0.34–0.40)**	0.69 (0.63–0.75)**
Clinical BMI								
< 18.5 18.5–24.9			0.26 (0.13–0.51)**	2.19 (1.49–3.24)**	0.16 (0.08–0.33)**	1.98 (1.32–2.97)*	0.12 (0.06–0.25)**	1.43 (0.94–2.16)
25–29.9			Ref	Ref	Ref	Ref	Ref	Ref
≥ 30			0.82 (0.77–0.87)** 0.95 (0.87–1.04)	1.00 (0.93–1.08) 0.83 (0.75–0.92)**	0.99 (0.92–1.06) 1.31 (1.19–1.44)**	1.27 (1.18–1.38)** 0.89 (0.80–0.99)*	1.14 (1.05–1.23)** 1.44 (1.30–1.59)**	1.50 (1.37–1.64)** 0.86 (0.76–0.97)*
Comorbidities								
No			Ref	Ref	Ref	Ref	Ref	Ref
1			3.31 (2.98–3.69)**	0.99 (0.89–1.10)	3.17 (2.83 -3.55)**	1.10 (0.99–1.23)	3.23 (2.87–3.65)**	1.25 (1.10–1.41)**
≥ 2			4.54 (4.08–5.04)**	2.31 (2.11–2.53)**	4.22 (3.79–4.71)**	2.50 (2.27–2.75)**	3.70 (3.30–4.15)**	2.15 (1.92–2.40)**
Lifestyle								
Smoking								
No					Ref	Ref	Ref	Ref
Former					0.40 (0.37–0.43)**	1.12 (1.03–1.22)*	0.35 (0.32–0.38)**	1.30 (1.18–1.43)**
Yes					1.25 (1.13–1.37)**	2.09 (1.87–2.34)**	0.94 (0.85–1.04)	1.53 (1.35–1.75)**
Alcohol								
No					Ref	Ref	Ref	Ref
Former					1.49 (1.25–1.77)**	2.67 (2.36–3.02)**	1.11 (0.93–1.32)	1.73 (1.50–1.99)**
Yes					2.29 (2.11–2.48)**	0.76 (0.69 -0.83)**	2.65 (2.43 – 2.88)**	0.92 (0.84 – 1.02)
Physical activity (MVPA/week)								
Lowest (0–6)					Ref	Ref	Ref	Ref
Middle (6.01–12.05)					1.33 (1.23–1.44)**	0.81 (0.74 -0.88)**	1.55 (1.42 – 1.69)**	0.99 (0.90–1.10)
Highest (12.06–49)					0.82 (0.76–0.88)**	0.87 (0.81 – 0.95)*	0.91 (0.83 – 0.98)*	1.00 (0.91–1.10)
Psychosocial								
Fatigue							1.04 (1.03–1.04**)	1.05 (1.05–1.06)**
Anxiety							1.11 (1.09–1.12)**	1.14 (1.12–1.16)**
Depressive symptoms							1.04 (1.02–1.05)**	1.11 (1.10–1.13)**
BIPQ cognition							0.99 (0.98–0.99)**	1.02 (1.02–1.03)**
BIPQ emotion							1.05 (1.04–1.06)**	0.99 (0.98–1.00)
*Pseudo R2*	0.05		0.07		0.12		0.21	

^*^p-value < 0.05, **p-value < 0.001 for adjusted odds ratios (AOR) where the reference category is indicated in the table or, for the continuous psychosocial variables AORs are for one unit change

## Results

### Study population

**Fig. 1:**
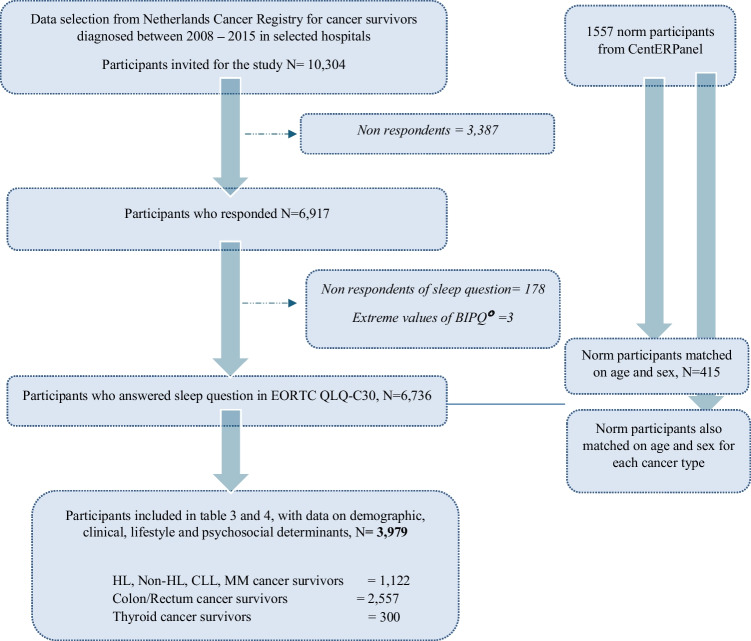
Flow chart of the study population. * Values of BIPQ exceeding highest total score of 80. HL = Hodgkins Lymphoma, CLL = Chronic Lympocytic Leukemia, MM=Multiple Myeloma

Of the 10,304 invited cancer survivors, 6917 responded (67%) of whom 6736 (65%) responded to the sleep question (Fig. 1). Based on these 6736 cancer survivors, a total of 415 normative individuals that matched on age and sex were extracted from the pool of 1557 individuals. The mean age of the normative population, cancer survivors who responded to the sleep question (respondents), and non-respondents was 66 years, while non-respondents were more often women (54%) compared to respondents (46%; Table 4). More than half of the respondents had secondary education (61%), were retired (78%), and were married (77%). The largest included cancer group was colorectal cancer (38%), and more than half of the respondents had surgery as their primary treatment (56%).

**Table 4:** Demographic, lifestyle, clinical, and psychosocial characteristics of study population, including the normative population, cancer survivors, and cancer survivors who did not respond to the study questionnaire (*n* = 3387) or did not respond to the sleep question (*n* = 178)

Determinants (*N*, %)	Normative population (n = 415)	Cancer survivor respondents (*n* = 6736)	Cancer survivor non-respondents (*n* = 3565) (3387 + 178)	Effect size^a^ (*p*-value) (Norm vs. survivors)	Effect size^a^ (*p*-value) (Respondents vs. non-respondents)
AGE (mean, SD in years)	66.1 (± 12.4)	66.2 (± 11.9)	66.1 (± 14.6)	NA	− 0.004 (0.827)
Sex				NA	− 0.059 (< 0.001)
Male	225 (54%)	3650 (54%)	1709 (48%)		
Female	190 (46%)	3086 (46%)	1854 (52%)		
Education			NA	0.205(< 0.001)	NA
Low (less then high school)	23 (5%)	1191 (18%)			
Secondary (high school)	220 (53%)	4087 (61%)			
High (bachelors or masters)	172 (42%)	1370 (21%)			
*Missing*		88			
Working status	NA		NA	NA	NA
No		5006 (78%)			
Yes		1451 (22%)			
*Missing*		279			
Marital status	NA		NA	NA	NA
Divorced/separated/widowed		1541 (23%)			
Married		5127 (77%)			
*Missing*		68			
Smoking				0.164 (< 0.001)	0.001 (0.994)
No	144 (34%)	1706 (34%)	32 (34%)		
Former	206 (49%)	2698 (54%)	51 (54%)		
Yes	65 (15%)	617 (12%)	12 (12%)		
*Missing*		1715	3468		
Alcohol				0.208 (< 0.001)	0.051 (< 0.002)
No	69 (16%)	1198 (26%)	36 (40%)		
Former	21 (4%)	406 (9%)	11 (12%)		
Yes	325 (77%)	3051 (65%)	43 (48%)		
*Missing*		2081	3473		
Physical activity	NA		NA	NA	NA
(MVPA/week)					
Lowest (0–6)		1333 (36%)			
Middle (6.01–12.05)		1177 (32%)			
Highest (12.06–49)		1179 (32%)			
*Missing*		3049			
BMI	NA			NA	0.015 (0.633)
< 18.5 (underweight) 18.5–24.9 (normal)		77 (1%) 2542 (38%)	2 (1%) 48 (33%)		
25–29.9 (overweight)		2876 (43%)	69 (48%)		
≥ 30 (obese)		1132 (17%)	26 (18%)		
*Missing*		109	3418		
Cancer type	NA			NA	0.125 (< 0.001)
Colorectal		2257 (38%)	1018 (29%)		
Prostate		686 (10%)	364 (10%)		
Borderline ovarian		83 (1%)	108 (3%)		
Ovarian		354 (5%)	249 (7%)		
Thyroid		300 (4%)	176 (5%)		
Endometrial		217 (3%)	78 (2%)		
Hodgkin lymphoma		209 (3%)	124 (3%)		
Non-Hodgkin lymphoma		1120 (17%)	615 (17%)		
CLL		281 (4%)	172 (5%)		
Multiple myeloma		255 (4%)	160 (4%)		
BCC/PCC		674 (10%)	499 (14%)		
Cancer stage	NA			NA	0.046 (< 0.001)
I		1622 (30%)	722 (28%)		
II		1868 (35%)	917 (36%)		
III		1310 (24%)	576 (22%)		
IV		609 (11%)	365 (14%)		
*Missing*		1327	983		
Primary treatment	NA			NA	
No/wait and see/watchful waiting		513 (8%)	370 (10%)		* − *0.047 (< 0.001)
Yes					
Surgery		3773 (56%)	1718 (48%)		0.074 (< 0.001)
Systemic		2355 (35%)	1151 (32%)		0.026 (0.006)
Radiation		1789 (27%)	768 (22%)		0.055 (< 0.001)
Hormonal		216 (3%)	151 (4%)		* − *0.026 (0.007)
Time since diagnosis	NA			NA	0.087 (< 0.001)
< 2 years		1937 (29%)	1266 (35%)		
≥ 2 to < 3 years		1340 (20%)	512 (5%)		
≥ 3 to < 5 years		1334 (20%)	641 (18%)		
≥ 5 years		2124 (31%)	1124 (32%)		
*Missing*		1	20		
Comorbidities	NA		NA	NA	NA
0		1683 (26%)			
1		1894 (30%)			
≥ 2		2849 (44%)			
*Missing*		310			
Fatigue	NA	21.3 (± 7.3)	NA	NA	NA
Distress	NA		NA	NA	NA
Anxiety		4.6 (± 3.8)			
Depressive symptoms		4.3 (± 3.7)			
Illness perception	NA		NA	NA	NA
Cognition		24.8 (± 8.4)			
Emotions		14.5 (± 5.5)			

^*^p < 0.05

*BCC/PCC* basal/squamous cell carcinoma, *CLL* chronic lymphocytic leukemia,* NA* not applicable or data not available

^*a*^Effect size = Cohens D for continuous variable and Phi Coefficient for categorical

### Prevalence of sleep problems compared to the normative population

The prevalence of sleep problems was higher in cancer survivors (17%, *n* = 1140) than in the normative population (11%, *n* = 46; Table 1) when matched based on all types of cancer survivors. When matched specifically based on each cancer type survivors of colorectal ovarian (highest), thyroid, Hodgkin’s lymphoma, non-Hodgkin’s lymphoma, chronic lymphocytic leukemia, multiple myeloma, and basal/squamous cell carcinoma cancer survivors reported higher prevalence of sleep problems compared to the matched normative population. The prevalence of sleep problems did not differ in survivors being longer post diagnosis.

### Determinants of sleep problems

The univariate analysis showed that demographic factors (sex, education, work, and having a partner), clinical factors (BMI, comorbidity), lifestyle factors (smoking, alcohol, and physical activity), and psychosocial factors (fatigue, anxiety, depressive symptoms, and illness perceptions) were associated with moderate and/or poor sleep (Table 2).

In the hierarchical regression, the full model (Table 3) showed that female cancer survivors (adjusted odds ratio (AOR) 2.26, 95% CI 2.06; 2.48) when compared to males, cancer survivors who had one comorbidity (AOR 1.25, 95% CI 1.10;1.41) and two or more comorbidities (AOR 2.15, 95% CI 1.92; 2.40) compared to cancer survivors with no comorbidity, cancer survivors who were former (AOR 1.30, 95% CI 1.18; 1.43), and current smokers (AOR 1.53, 95% CI 1.35;,1.75) compared to cancer survivors who never smoked, cancer survivors who were former alcohol drinkers (AOR 1.73, 95% CI 1.50; 1.99) compared to cancer survivors who did not drink alcohol, cancer survivors with fatigue (AOR 1.05, 95% CI 1.05; 1.06), anxiety (AOR 1.14, 95% CI 1.12; 1.16), depression (AOR 1.11, 95% CI 1.10; 1.13), and higher cognitive illness perception (AOR 1.02, 95% CI 1.02; 1.03) had a higher odds for sleep problems regardless of the included demographic factors. Whereas, cancer survivors with higher education (AOR 0.67, 95% CI 0.58; 0.76) compared to low education, cancer survivors having a partner (AOR 0.69, 95% CI 0.63; 0.75) compared to having no partner and cancer survivors who were obese (AOR 0.86, 95% CI 0.76; 0.97) compared to normal BMI showed to have a lower odds for sleep problems (Table 3). Working status and physical activity were not significantly associated with sleep problems when taking other covariables into account. However, in model 3 (with lifestyle determinants), cancer survivors with highest levels of physical activity compared to lowest levels of physical activity had a lower odds for sleep problems (AOR 0.91, 95% CI 0.83; 0.98). The full model showed a maximum change in *R*^2^ when compared to the previous models. This model showed a pseudo *R*^2^ of 0.21 compared to 0.05 for the starting model with only demographic determinants.

## Discussion

In this study, we found that the proportion of survivors with sleep problems was higher in cancer survivors when compared to the normative population. The prevalence of survivors with sleep problems differed across cancer types, with the highest prevalence in ovarian cancer survivors. The literature suggests that ovarian cancer survivors may feel stressed due to the high recurrence rate in combination with non-specific alarming symptoms (bloated or uncomfortable feeling a the abdomen) which may cause higher distress and post diagnosis biological changes leading to sleep problems [23]. The prevalence of sleep problems among cancer survivors in our study was lower when compared to previous studies that focused on single cancer types [8, 9, 14]. To the best of our knowledge, our study is one of the few that compares the prevalence of sleep problems in cancer survivors of various types with an age- and sex-matched normative population. A lower prevalence of sleep problems in our study could be attributed to the absence of breast cancer survivors in our study population which is one of the most frequent and researched type of cancer with higher numbers of survivors with sleep problems  [43–45].

We found several demographic factors such as female sex, lower education, and not having a partner, having a higher odds of experiencing sleep problems. These findings align with some previous studies, which also indicated phenomenon related to higher level of anxiety and fear of cancer progression in female cancer survivors associated with more sleep problems [1, 3, 6, 8, 15]. However, survivors with higher education and survivors who had a partner had a lower odds for sleep problems. These findings align with previous research attributing health literacy and engagement in interventions to improve sleep quality such as mind–body medicine among higher educated cancer survivors and the role of social support in patients with a partner on positive sleep outcomes [1, 15]. Additionally, we identified clinical factors BMI and presence of comorbidities as significant determinants of sleep problems. Consistent with the existing literature survivors with one or more comorbidities had a higher odds for sleep problems which may be attributed to various systemic complaints, symptoms, and drug interactions disturbing an uninterrupted sleep [3, 20]. In contrast to previous studies, we found no association between primary treatment and sleep problems [46]. Previous studies showed that cancer survivors who underwent chemotherapy as primary treatment had a higher odds for sleep problems [14, 17]. A reason that primary treatment was not associated with sleep problems in our study might be that other determinants which are strongly associated with sleep problems (e.g., psychosocial factors) confound the association between primary treatments and sleep problems. A second reason might be that treatment in a diverse cancer population might be heterogeneous. For instance, chemotherapy might include different agents and different regiments across cancer types. Furthermore, in our analyses, we could not adjust for sleep medication because these data were not available. Considering lifestyle factors and consistent with previous research, we found current smoking cancer survivors and both former smoking and former alcohol drinking cancer survivors to have a higher odds for sleep problems. Furthermore, cancer survivors who experienced fatigue were anxious and or depressive, and had threatening cognitive illness perception also had a higher odds for sleep problems which was similar to findings from previous studies that suggested hormonal imbalances following treatment, treatment side effects, and psychological distress contribute to sleep problems [9, 13, 15]. Psychological distress presenting as anxiety and or depression due to fear of death, recurrence, and poor prognosis may impact sleep quality [4, 8, 9, 21]. The psychosocial determinants, particularly fatigue, anxiety, depression, and illness perception contributed significantly to the variability in sleep quality among cancer survivors. Survivors with highest MVPA scores for physical activity had a lower odds for sleep problems compared to survivors with lowest MVPA scores before adjusting for psychosocial variables, which may be plausible as physical activity is also influencing for instance, anxiety, fatigue, depression, and illness perception.

The pseudo-R2 was very small indicating that there are more or more important determinants of sleep problems for which we have no data and thus could not include in the model (e.g., sleep medication).

There were several strengths of this study. First, the large sample size, that included various types of cancers and determinants, gave us enough statistical power to investigate differences across cancer types. Second, the inclusion of an age- and sex-matched normative population as a comparison group allowed for a fair comparison between each cancer type and the normative population. Third, a notable strength was the use of registry-based diagnosis ensuring verified, up to date and accurate clinical data. Few previous studies have employed such robust data collection methods on clinical variables.

However, there were also certain limitations to our study. First, the cross-sectional design does not allow drawing conclusions on the causality between sleep problems, fatigue, anxiety, depression, and illness perception—significant determinants in the final model. We cannot assert that higher fatigue, anxiety, and/or depression causes sleep problems, as the reverse relation could also be the case. Moreover, these symptoms are known to cluster and might be interrelated in a more complex manner than can be identified even with longitudinal data. Second, we did not have data of survivors of all cancer types (e.g., no lung and breast cancer), various domains of sleep quality, and the use of sleep medication among cancer survivors. This might have attenuated the current prevalence and point estimates of sleep problems when compared to good sleepers. Studies have shown a prevalence of sleep problems of up to 56% in lung cancer survivors and 38% in breast cancer survivors [43, 47]. The absence of these cancer types in our analysis might explain the lower overall prevalence of sleep problems in the present study. Additionally, since no data about sleep medication was available, it is possible that cancer survivors with sleep problems using sleep medication reported not experiencing trouble sleeping. Another limitation might be that the data were collected between May 2008 and April 2015. However, the problem of sleep problems after cancer is still as prevalent today as it was then. As the included determinants of sleep problems are largely unrelated to changes in healthcare and treatment, we believe that the results of our study is still of great value to this field of research. Furthermore, we used the sleep quality scale of the EORTC QLQ-C30, a scale only consisting of a single-item. Although a single-item measure in calculating prevalence estimates likely limits the accuracy, earlier studies showed that the sleep item of the EORTC QLQ-C30 was highly correlated (*r* = 0.71) with the total score of the Pittsburgh Sleep Quality Index (PSQI), a multiple-item questionnaire [33, 34]. We also used a unidimensional measure of fatigue that may have led to an underestimation of the prevalence of fatigue, which may dilute the effect size. Moreover, it hampers the ability to assess the impact of different dimensions of fatigue. Lastly, the reliance on self-reported data could have potentially biased the estimates of certain determinants. We attempted to overcome over- and underestimation by categorizing physical activity and BMI.

The clinical implications of this study suggest that early screening for sleep problems by healthcare professionals in clinical settings is warranted. Patients can be supported by focusing on improving various modifiable lifestyle factors after cancer treatment that might help to prevent or diminish sleep problems. These factors include increasing physical activity and decreasing smoking and alcohol consumption. As fatigue, depression, and illness perception seems related, it might be possible that increasing physical activity also has a positive effect on these psychological factors besides sleep problems. Patients can be referred to interventions that address psychological factors such as cognitive behavioral therapies, Mindfulness-Based Stress Reduction Programs, or yoga [4, 11, 48, 49]. Research implications include future prospective cohort studies with other cancer types, exploring domains of sleep quality and investigating the effects of the interventions that address sleep problems.

In conclusion, cancer survivors exhibit a higher prevalence of sleep problems compared to a normative population, with the highest prevalence in ovarian cancer survivors. Female cancer survivors, cancer survivors who do not have higher education, cancer survivors without a partner, with comorbidities, former and current smokers, former alcohol drinkers, cancer survivors having low physical activity levels, high fatigue levels, elevated anxiety, depression, and cognitive illness perception levels were more prone to have sleep problems. Notably, psychosocial determinants add to the variability in sleep problems of cancer survivors. Health care professionals need to be aware of the high prevalence of sleep problems in cancer survivors and should pay attention to these problems during follow-up visits even years after diagnosis.

## Data Availability

After publication, de-identified individual participant data and syntax files will be shared upon request, for scientific purposes, after approval of the proposal, with a signed data access agreement and with investigator support. Contact information can be found at the PROFILES registry webpage: https://www.profilesregistry.nl/contact/.
